# Hypomethylation of smoking-related genes is associated with future lung cancer in four prospective cohorts

**DOI:** 10.1038/ncomms10192

**Published:** 2015-12-15

**Authors:** Francesca Fasanelli, Laura Baglietto, Erica Ponzi, Florence Guida, Gianluca Campanella, Mattias Johansson, Kjell Grankvist, Mikael Johansson, Manuela Bianca Assumma, Alessio Naccarati, Marc Chadeau-Hyam, Ugo Ala, Christian Faltus, Rudolf Kaaks, Angela Risch, Bianca De Stavola, Allison Hodge, Graham G. Giles, Melissa C. Southey, Caroline L. Relton, Philip C. Haycock, Eiliv Lund, Silvia Polidoro, Torkjel M. Sandanger, Gianluca Severi, Paolo Vineis

**Affiliations:** 1 Molecular end Epidemiology Unit, HuGeF, Human Genetics Foundation, Torino 10126, Italy; 2 Unit of Cancer Epidemiology, Citta' della Salute e della Scienza Hospital-University of Turin, Center for Cancer Prevention, Via Santena 7, Torino 10126, Italy; 3 Inserm (Institut National de la Santé et de la Recherche Médicale), Centre for Research in Epidemiology and Population Health, U1018, Team 9, 114 rue Edouard Vaillant, Villejuif 94805, France; 4 Paris-South University, Villejuif 91450, France; 5 Department of Genetic Epidemiology, Cancer Epidemiology Centre, Cancer Council of Victoria, Melbourne, Victoria 3004, Australia; 6 School of Population and Global Health, Centre for Epidemiology and Biostatistics, University of Melbourne, Melbourne, Victoria 3010, Australia; 7 MRC-PHE Centre for Environment and Health, Department of Epidemiology and Biostatistics, School of Public Health, Imperial College London, Norfolk Place, London W2 1PG, UK; 8 Genetic Epidemiology Division, International Agency for Research on Cancer, Lyon 69008, France; 9 Department of Biobank Research, Umeå University, Umeå SE—90187, Sweden; 10 Department of Radiation Sciences, Umeå University, Umeå SE—90187, Sweden; 11 Department of Molecular Biotechnology and Health Sciences, Università di Torino, Torino 10126, Italy; 12 Division of Epigenomics and Cancer Risk Factors, DKFZ—German Cancer Research Center, Heidelberg 69121, Germany; 13 Division of Cancer Epidemiology, DKFZ—German Cancer Research Center, Heidelberg 69121, Germany; 14 Translational Lung Research Center Heidelberg, Member of the German Center for Lung Research (DZL), Heidelberg 69120, Germany; 15 Division of Cancer Research and Epigenetics, Department of Molecular Biology, University of Salzburg, Salzburg 5020, Austria; 16 Department of Medical Statistics, London School of Hygiene and Tropical Medicine, London WC1E 7HT, UK; 17 Genetic Epidemiology Laboratory, University of Melbourne, Melbourne, Victoria 3010, Australia; 18 MRC Integrative Epidemiology Unit, School of Social and Community Medicine, University of Bristol, Bristol BS8 2BN, UK; 19 Department of Community Medicine UiT–The Arctic University of Norway, Tromso 9019, Norway

## Abstract

DNA hypomethylation in certain genes is associated with tobacco exposure but it is unknown whether these methylation changes translate into increased lung cancer risk. In an epigenome-wide study of DNA from pre-diagnostic blood samples from 132 case–control pairs in the NOWAC cohort, we observe that the most significant associations with lung cancer risk are for cg05575921 in *AHRR* (OR for 1 s.d.=0.37, 95% CI: 0.31–0.54, *P*-value=3.3 × 10^−11^) and cg03636183 in *F2RL3* (OR for 1 s.d.=0.40, 95% CI: 0.31–0.56, *P*-value=3.9 × 10^−10^), previously shown to be strongly hypomethylated in smokers. These associations remain significant after adjustment for smoking and are confirmed in additional 664 case–control pairs tightly matched for smoking from the MCCS, NSHDS and EPIC HD cohorts. The replication and mediation analyses suggest that residual confounding is unlikely to explain the observed associations and that hypomethylation of these CpG sites may mediate the effect of tobacco on lung cancer risk.

## Abstract (web summary)

Smoking tobacco is known to alter DNA methylation. Here, the authors show that hypomethylation of smoke-related genes is associated with future increase in lung cancer risk.

## Graphical abstract

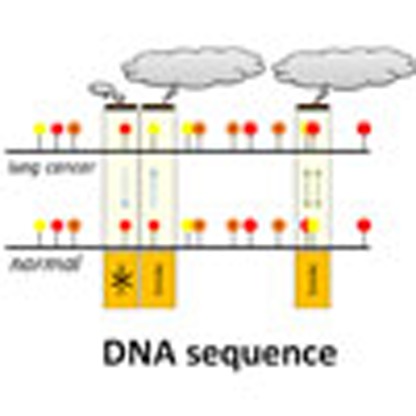

DNA methylation has recently emerged as an important marker of current and past smoking habits^1^,^2^,^3^,^4^,^5^,^6^,^7^,^8^,^9^. Smoking is a leading cause of death worldwide^10^,^11^ and has been identified as a major risk factor for several diseases including cancer^12^,^13^, cardiovascular^14^,^15^ and respiratory diseases^16^,^17^. The carcinogenic effect of tobacco smoking persists for decades after smoking cessation, and former smokers remain at increased risk of lung cancer for 20 years or longer^18^,^19^,^20^.

Using an epigenome-wide methylation study approach, we previously demonstrated that tobacco smoking alters DNA methylation patterns, particularly in CpG sites of the *AHRR* and *F2RL3* genes^7^. These results have been extensively replicated by other studies^1^,^2^,^3^,^4^,^5^,^6^,^8^. In particular, our previous study of 1,000 healthy subjects from the EPIC and Norwegian Women and Cancer (NOWAC) cohorts indicated that smokers had 19% lower methylation levels at the *AHRR* CpG site cg05575921 compared with never-smokers. We also found that one set of specific methylation markers showed a gradual reversal of methylation levels from those typical of current smokers to those of never-smokers, whereas other smoking-related CpG sites' methylation markers remained stable more than 30 years after quitting^9^. These findings are also consistent with other recent studies reporting methylation levels in smoking-related CpG loci in former smokers to vary based on their time since quitting^21^,^22^.

Although these previous studies have provided convincing evidence of an association between tobacco exposure and methylation of specific CpG sites, it is not known whether methylation levels at some of these sites translate into increased risk of smoking-related cancers, such as lung cancer. Here, we present the results of an epigenome-wide methylation study based on methylation detection using Illumina Infinium HM450 on DNA extracted from pre-diagnostic blood of 132 pairs of lung cancer cases and controls from the NOWAC cohort (discovery set). We replicated the findings in three prospective studies, the Melbourne Collaborative Cohort Study (MCCS; 367 cases and 367 matched controls), the Northern Sweden Health and Disease Study (NSHDS; 234 cases and 234 matched controls) and the EPIC Heidelberg Study (EPIC HD; 63 cases and 63 matched controls; validation sets), with adjustment for smoking habits. To our knowledge, this is the first study performing a genome-wide methylation analysis to evaluate the importance of epigenetic alterations in peripheral blood DNA to lung cancer aetiology.

## Results

### Discovery set

Incident lung cancer cases in the discovery set (NOWAC) were identified through linkage with the Cancer Registry of Norway, with virtually complete coverage. In the nested case–control study, lung cancer cases were diagnosed on average 3.88 years after recruitment (range: 0.29–7.92 years) and the mean age at diagnosis was 56 years (range: 47–64 years). The odds ratio for lung cancer was 7.38 for former and current smokers grouped together (95% confidence interval (CI) 3.99–16.66), 6.16 (95% CI: 2.65–15.13) for former smokers and 10.13 (95% CI: 4.56–24.23) for current smokers.

Table 1 shows the top-ranked CpG sites for the locus-by-locus epigenome-wide risk analysis, and includes all CpG sites with Bonferroni-corrected *P*-values below 0.05. All top-ranked CpGs showed inverse associations with risk, indicating hypomethylation in cancer cases. Supplementary Table 1 shows the main information about involvement in cancer pathways for the probes listed in Table 1: for all the CpGs except two (cg02451831 and cg03898802) there is evidence of involvement in cancer pathways. CpGs in the *AHRR* and *F2RL3* genes displayed the most significant associations with risk consistent with previous observations of smoking being associated with reduced methylation in healthy subjects^1^,^2^,^3^,^4^,^5^,^6^,^7^,^8^,^9^. In the following analyses, we exclusively focus on these two genes from Table 1, because they are the only ones strongly associated with smoking. In particular, the cg05575921 probe in the *AHRR* gene emerged as the CpG site most strongly associated with both tobacco exposure^9^ and lung cancer risk (odds ratio (OR) for lung cancer per 1 standard deviation (s.d.) of beta: 0.37, 95% CI: 0.31–0.54, *P*-value=3.33 × 10^−11^). Sensitivity analyses excluding cases with time from blood collection to diagnosis of <2 years showed no significant differences in effect estimates (OR: 0.36, 95% CI: 0.27–0.52 for cg05575921 and OR: 0.40, 95% CI: 0.29–0.56 for cg03636183). Supplementary Table 2 shows the results of the analyses stratified by time to diagnosis (less and more than 5 years). Associations were slightly stronger for <5 years to diagnosis but these were unlikely to reflect reverse causation as they were also evident for more than 5 years to diagnosis.

Table 2 shows the results for the probes associated with cancer risk in the *AHRR* and *F2RL3* genes after adjustment for smoking (for example, smoking status coded as never, former, current): the overall association remained basically unchanged (OR for 1 s.d.=0.39, 95% CI: 0.24–0.61, *P*-value=2.55 × 10^−5^ for cg05575921 and OR for 1 s.d.=0.51, 95% CI: 0.35–0.73, *P*-value=4.19 × 10^−4^ for cg03636183).

### Validation sets

To validate our results arising from the NOWAC study, we analysed the cg05575921 and cg03636183 probes in three independent samples: a case–control study nested within MCCS including 367 case–control pairs, a case–control study nested within the NSHDS including 234 case–control pairs and a case–control study nested within the EPIC HD cohort, including 63 case–control pairs, all of which were matched on smoking status (see Methods for details).

Consistent with the results from the NOWAC study, methylation levels in the MCCS, NSHDS and EPIC HD studies were clearly inversely associated with lung cancer risk for both the cg05575921 and cg03636183 CpG sites. The overall OR estimates were slightly weaker in MCCS than in NOWAC (OR: 0.62, 95% CI: 0.50–0.78, *P*=2.91 × 10^−5^ for cg05575921 and OR: 0.70, 95% CI: 0.58–0.85, *P*=2.21 × 10^−4^ for cg03636183), but more comparable in NOWAC to NSHDS and EPIC HD (OR: 0.42, 95% CI: 0.30–0.58, *P*=2.06 × 10^−7^ for cg05575921 and OR: 0.61, 95% CI: 0.47–0.79, *P*=1.56 × 10^−4^ for cg03636183 in NSHDS; OR: 0.45, 95% CI: 0.22–0.92, *P*=2.95 × 10^−2^ for cg05575921 and OR: 0.62, 95% CI: 0.38–1.04, *P*=7.02 × 10^−2^ for cg03636183 in EPIC HD; Table 2). We note that some attenuation of the overall NOWAC OR estimates is expected as MCCS, NSHDS and EPIC HD studies were matched by smoking status.

### Risk prediction model for lung cancer

We applied to the NOWAC cohort a prediction model including smoking status (coded as never, former, current) and methylation as a covariate. This was not feasible for the other cohorts because of matching by smoking. The area under the curve (AUC) of the model increased from 0.71 to 0.76 when adding *AHRR-*methylation and *F2RL3-*methylation as categorical variables (above or below the median) and to 0.78 when adding the two as continuous variables.

### Lung cancer risk by categories of smoking exposure

To further evaluate the associations of the cg05575921 and cg03636183 CpG sites with lung cancer risk, we conducted stratified risk analysis by categories of smoking status. We found little support for an association being present for never smokers for either CpG site, and the associations were clearly influenced by smoking. A notable observation regarding ever smokers was that the association appeared to be stronger for former smokers than for current smokers. For instance, in the NOWAC study, the OR for the cg05575921 site was 0.23 (95% CI: 0.10–0.56) for former smokers, and 0.46 (95% CI: 0.24–0.88) for current smokers. This pattern was evident also in MCCS, NSHDS and EPIC HD for both the cg05575921 and cg03636183 CpG sites (Table 2).

### Methylation of *AHRR* and *F2RL3* genes in former smokers

**Figure 1:**
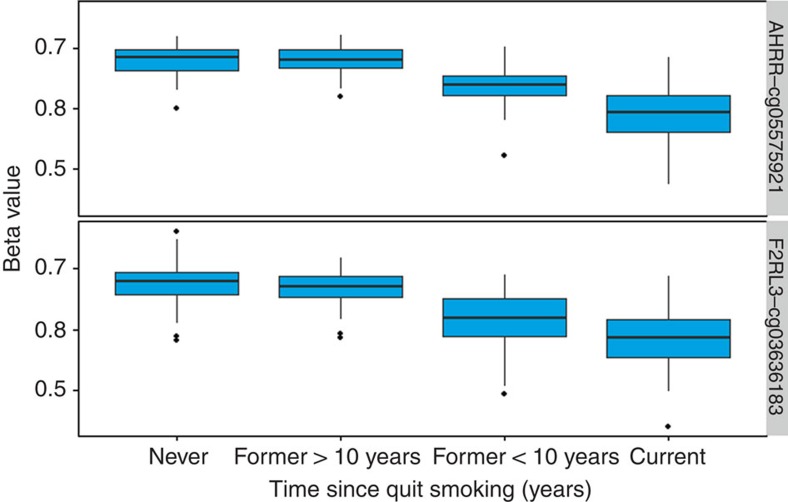
NOWAC cohort. Associations between smoking cessation (years since quitting on horizontal axis) and methylation levels (vertical axis).

The associations between smoking cessation and the mean methylation levels in the cg05575921 probe (*AHRR* gene) and the cg03636183 probe (*F2RL3* gene) in NOWAC are shown in Fig. 1. After smoking cessation, methylation levels increase and after 10 years since quitting appear to approach those of never smokers. This is consistent with the well-documented observation that the risk of lung cancer decreases substantially after smoking cessation.

**Figure 2:**
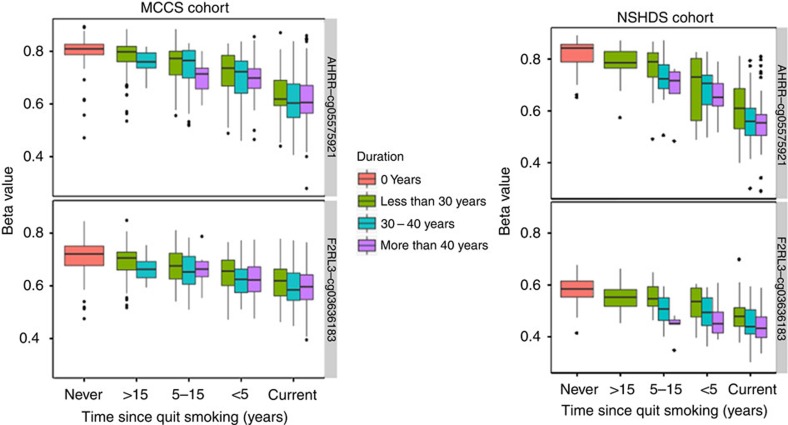
MCCS and NSHDS cohorts. Associations between duration of smoking and time since smoking cessation and methylation levels in *AHRR*-cg05575921 and *F2RL3*-cg03636183.

The effect of smoking (never versus former versus current; time since quitting smoking; smoking duration) on methylation beta levels for cg05575921 and cg03636183 in MCCS and in NSHDS are shown in Fig. 2. Similar to what we observed in NOWAC (Fig. 1), in MCCS and NSHDS, methylation levels in current smokers were lower than methylation levels in never smokers and in former smokers the levels approached those of never smokers with increasing time since cessation.

### Comparison of the study groups

Supplementary Table 3 shows a summary of the key characteristics of the study groups. The limitation to a single gender in NOWAC prevented us from making straightforward comparisons between the estimated associations and from investigating differences in lung cancer risk between genders. On the other hand, matching by smoking in MCCS, NSHDS and EPIC HD did not allow us (i) to investigate further the role of methylation as a mediator of the association between smoking and cancer in these cohorts and (ii) to test interactions between smoking variables such as duration or dose. A future goal will be to repeat the analysis in unrestricted population cohorts.

### Correlation between methylation and expression

We investigated the correlation between methylation and expression of the two relevant probes using two different sources of data: TCGA (http://cancergenome.nih.gov/) and HapMap (http://hapmap.ncbi.nlm.nih.gov/). In TCGA, we focused on expression (RNA-Seq experiments) and methylation (Illumina HumanMethylation450 BeadChip) of samples of normal tissue (i) from 21 lung adenocarcinoma cases (LUAD—21 methylation-expression pairs) and (ii) from 8 lung squamous cell carcinoma cases (LUSC—8 methylation-expression pairs). *AHRR-*probe methylation seems to be significantly inversely correlated with *AHRR* expression in LUAD and the same trend was found in LUSC (Pearson's correlation coefficient=−0.66, *P* value<0.01 in LUAD; Pearson's correlation coefficient=−0.43, *P* value=0.29 in LUSC). *F2RL3-*probe methylation did not show a statistically significant methylation-expression correlation. Regarding HapMap, we focused on expression (RNA-Seq experiments) and methylation (Illumina HumanMethylation27 BeadChip) data from lymphoblastoid cell lines of 69 HapMap Yoruba individuals. In this case, only the *F2RL3-*probe is present on the platform and its methylation seems to be significantly inversely correlated with *F2RL3* expression (Pearson's correlation coefficient=−0.28, *P* value<0.01).

### Mediation analysis

Although the results described above from the analysis of a discovery set and three validation sets seem to provide evidence that hypomethylation of the cg05575921 and cg03636183 probes is associated with both tobacco exposure and lung cancer risk, the key question is whether their hypomethylation is involved in the causal pathway, or whether they are simply epiphenomena of smoking habits (that is, the association of DNA methylation with lung cancer risk is confounded by smoking). To bring some clarity to this question, we used mediation analysis to quantify the amount by which cg05575921 (*AHRR* gene) and cg03636183 (*F2RL3* gene) methylation might mediate the effect of smoking on lung cancer incidence. This was performed for the NOWAC study as such an analysis was not possible for the MCCS, NSHDS or EPIC HD because of matching by smoking status.

**Figure 3:**
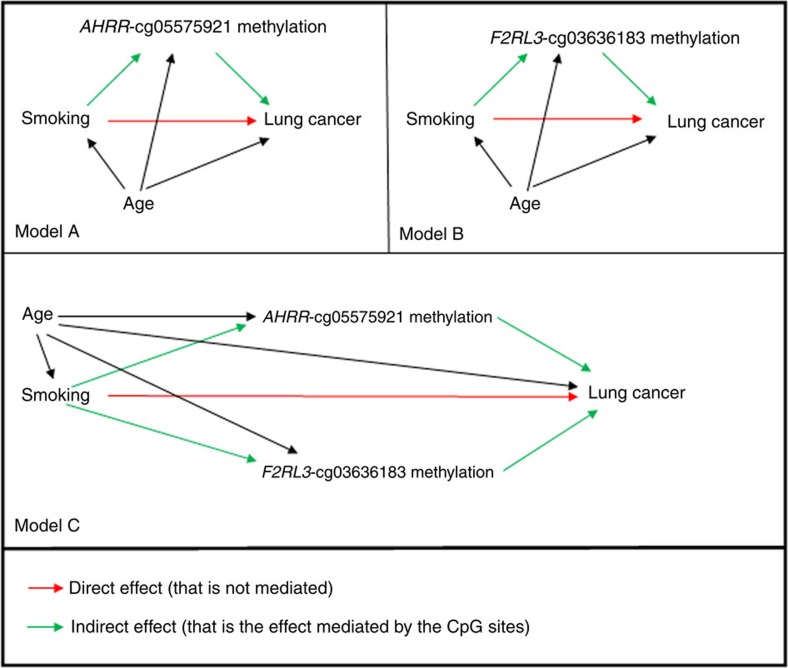
Mediation analysis: graphical representation. In model A, the percentage of the effect mediated by *AHRR*-cg05575921 is ∼31% of the total effect of smoking on lung cancer risk, whereas in model B, the percentage mediated by *F2RL3-*cg03636183 is ∼32%. The joint mediation effect of these two CpGs is 37% if the two mediators are included together in the model with separate pathways (model C).

We detected statistically significant results for both components of mediation analysis, the natural direct effect (NDE) of smoking on lung cancer (that is, not mediated) and the natural indirect effect (NIE, that is, the effect mediated by the methylated probe(s)), the two together making up the total causal effect (TCE; see Methods and Table 3, where the underlying identifying assumptions are also stated). The proportion of the smoking-induced risk increase explained by cg05575921 *AHRR*-probe was found to be ∼31% (0.31, 95% CI: 0.18–0.46) and 32% (0.32, 95% CI: 0.20–0.53) for the cg03636183 *F2RL3*-probe. Considering the two genes together, their methylation appeared to mediate ∼37% (0.37, 95% CI: 0.19–0.66) of the total effect of smoking on lung cancer odds (Fig. 3 and Table 3). The results of mediation analysis were similar when we included the mean methylation of a group of ten *AHRR* (cg05575921, cg03991871, cg12806681, cg23916896, cg01899089, cg26703534, cg14817490, cg25648203, cg21161138 and cg24090911) and two *F2RL3* probes (cg03636183 and cg04259305) located in the body of the gene and significantly associated with lung cancer after false discovery rate correction (data not shown). In conclusion, this analysis suggests (i) that methylation of the smoking-related *AHRR* and *F2RL3* CpG sites might be relevant to lung cancer aetiology and (ii) would explain approximately one-third of the risk increase induced by tobacco exposure.

## Discussion

Tobacco smoking is one of the most important carcinogenic exposures, and continuing smokers experience up to 25% lifetime risk of developing a smoking-related cancer—particularly lung cancer—yet the underlying mechanisms by which tobacco carcinogens act on lung cells have been elusive. Mutations, cell proliferation and selection have been hypothesized as complementary mechanisms^23^,^24^. Epigenetics has recently emerged as a promising field to illuminate carcinogenetic mechanisms^23^ and we have previously shown that smoking is associated with hypomethylation in CpGs of key genes^9^. Here, we present data from four prospective cohort studies that convincingly demonstrate that hypomethylation in specific CpG sites of the *AHRR* and *F2RL3* genes is associated with increased risk of subsequent lung cancer. Although we detected 11 CpG sites in the discovery set that were associated with lung cancer, we selected the *AHRR* and *F2RL3* genes because of their strong association with smoking found in previous studies, and because our aim was to test whether methylation may feature in the pathway from smoking to lung cancer. *AHRR* is the repressor of the aryl hydrocarbon receptor, a key regulator of the relationships between the cell and the external environment, including the effects of stressors such as dioxins and polycyclic aromatic hydrocarbons (that are contained in tobacco smoke)^25^. *AHRR* is expressed in all tissues, where it controls cell proliferation and apoptosis; it is upregulated and epigenetically modified in lung alveolar macrophages of smokers^1^. We have previously investigated the lung tissue of smokers and non-smokers: methylation levels in the *AHRR* gene probes were significantly lower (*P*<0.001) with a concurrent increase in *AHRR* expression (*P*=0.005) in the lung tissue of current smokers compared with non-smokers^7^. This was further validated in a mouse model of smoke exposure^7^.

*F2RL3* is also a functionally relevant gene. It encodes the protease-activated receptor-4, which has been suggested to be involved in the pathophysiology of both cardiovascular and neoplastic diseases^26^. A recent study reported that hypomethylation of *F2RL3* is predictive of total mortality and the authors suggested that the adverse health effects of smoking might be mediated in part by pathways related to *F2RL3* methylation^26^.

The main question arising from our previous studies of healthy subjects was whether methylation changes in the *AHRR* and *F2RL3* genes are causally involved in lung cancer aetiology by mediating the risk induced by tobacco smoking. Although it is not possible to fully answer this question based on our data, our results are consistent with the notion of a mediating role. We have observed (i) that data from multiple independent study populations have conclusively established an association between tobacco smoking and *AHRR* and *F2RL3* methylation, and (ii) that these methylation sites are also associated with lung cancer risk after adjustment for smoking habits and with careful mediation analysis. Although it is possible that residual confounding from tobacco smoking might still explain the association with risk, we note that the attenuation in OR estimates when adjusting for smoking is negligible in all four studies. Should residual confounding from tobacco smoking explain our observed associations, we would expect a notable attenuation of OR estimates in adjusted risk models. In addition, the observation that smoking-associated hypomethylation in these specific CpG sites is reversible following smoking cessation is compatible with the gradual decrease in lung cancer risk that former smokers experience. A full evaluation of the causal relevance of *AHRR* and *F2RL3* methylation in lung cancer aetiology requires additional investigations, such as a Mendelian randomization analysis of a sufficiently powered study^27^. Hypomethylation of certain CpG sites/genes, which extends beyond smoking cessation for several years, as observed for the two probes identified in this study, might be more closely associated with lung cancer risk than transient hypomethylation. In previous analyses of healthy subjects^9^, we generally observed a relatively rapid reversal of smoking-related methylation changes, but for a group of probes including cg05575921 and cg03636183 reversal is slower or not apparent even after decades. A larger study is required to evaluate whether reversal of methylation alterations in cg05575921 and cg03636183 occurs at the same rate as the decrease in the risk of lung cancer in former smokers. Also, future prediction models will be built based on a larger number of cohorts not matched by smoking habits (work in preparation). In the present study, we were able to build such a model only for the NOWAC cohort, and there was a modest increase in prediction (AUC changing from 0.71 to 0.78 when methylation information was added).

Hypomethylation persists in some CpG sites for much longer than the average half-life of circulating white-blood cells, suggesting that stem cells (in the bone marrow in the case of white blood cells, and hypothetically also in the lung^1^) may preserve a ‘memory' of past exposures in the form of a greater proportion of unmethylated CpG sites versus methylated CpG sites. We speculate that exposure to toxic agents leads to clonal expansion of cells that are hypomethylated in CpGs of genes involved in activation of a pathway reactive to environmental insults, and this imbalance in the proportion of methylated DNA in stem cells persists, remaining mitotically stable through subsequent cell divisions.

The association of hypomethylation at the two selected CpG sites with lung cancer was nominally stronger for former than for current smokers in all our studies but this observation could be due to chance or residual confounding by factors related or unrelated to smoking.

In conclusion, our study shows that smoking-induced hypomethylation in the *AHRR* and *F2RL3* genes is associated with important risk increases of subsequent lung cancer, and indicates that these specific methylation alterations may mediate the carcinogenic effect of tobacco exposure in lung cancer aetiology.

## Methods

### Discovery set

Lung cancer cases and matched controls were identified within the Norwegian *NOWAC* longitudinal cohort. The biobank of the NOWAC cohort was collected in the years 2003–2006. Random samples of Norwegian women were mailed a letter of information with an invitation to receive equipment for blood sampling at the local doctor or other institutions. Those who filled in the eight-page questionnaire and accepted the invitation to donate blood received some months later equipment for blood drawing together with a two-page questionnaire with information on date, lifestyle factors and so on. Around 50,000 women returned by over-night mail two tubes of blood to the Institute of Community Medicine at UiT–The Artic University of Norway. Upon arrival, the citrate glass tube was centrifuged and buffy-coat and plasma frozen immediately at −80° together with a PAXgene tube. All participants gave informed consent. The study was approved by the Regional Committee for Medical and Health Research Ethics in North Norway. Data storage and linkage to the National Cancer Registry of Norway were approved by the Norwegian Data Inspectorate; follow-up identified 132 eligible cases of lung cancer by 2011. For each case, one control with adequate blood samples was selected matched on time since blood sampling and year of birth in order to control for effects of storing time and ageing. The cases and the controls were kept together through all later laboratory procedures in order to reduce any batch effects.

### Validation sets

The MCCS is a prospective cohort study of 41,514 volunteers (24,469 women) aged between 27 and 76 years at baseline (99.3% of whom were aged 40–69 years)^28^. The MCCS study protocol was approved by the Cancer Council Victoria's Human Research Ethics. At baseline attendance, in 1990–1994, participants completed questionnaires that measured demographic characteristics and lifestyle factors including diet. Height and weight were directly measured and a blood sample was collected and stored. For a large proportion of individuals (75%), only dried blood spots on Guthrie cards were available while for others buffy coat or lymphocyte samples were available. A total of 533 incident cases of lung cancer identified through linkage with the State and National Cancer Registry was diagnosed during follow-up up to the end of 2011. A total of 367 cases remained available after excluding cases (i) diagnosed after the age of 80 years; (ii) with no biospecimen available; (iii) with a diagnosis of any cancer before blood draw or (iv) with no information on smoking status. The MCCS sample included 367 cases (159 adenocarcinomas, 33 large cell cancers, 73 squamous cancers and 49 small cell cancers) and 367 matched controls selected with a density sampling procedure. Matching variables included sex, date of blood collection (within 6 months), date of birth (within 1 year), country of birth (Australia and UK versus Southern Europe), type of biospecimen (lymphocyte, buffy coat and dried blood spot) and smoking status (never smokers; short-term former smokers: quitting smoking less than 10 years before blood draw; long-term former smokers: quitting smoking 10 years or more before blood draw; current light smokers: <15 cigarettes per day at blood draw; and current heavy smokers: 15 cigarettes or more at blood draw). In the sample, the mean time between blood draw and diagnosis was 9.38 years (s.d., 5years).

The NSHDS is an ongoing prospective cohort and intervention study intended for health promotion in the population of Västerbotten County in northern Sweden. The study was approved by the Umeå University Ethical Committee; details of the study population have been published previously^29^. Briefly, study participants were recruited to the NSHDS in the context of the Västerbotten Intervention Project, which was initiated in 1985 to advocate a healthy diet and lifestyle. All residents in the Västerbotten County were invited to participate in the project by attending a health check-up at 40, 50 and 60 years of age. At the health check-up, which was held at the local health-care centre, participants were asked to complete a self-administered questionnaire including various demographic factors such as education, smoking habits, physical activity and diet. In addition, height and weight were measured and participants were asked to donate a blood sample of 20 ml for future research. Incident lung cancer cases were identified through linkage to the regional cancer registry. Lung cancer cases were defined on the basis of the International Classification of Diseases for Oncology, Second Edition (ICD-O-2), and included all primary malignant cancers that are coded as C34.0-C34.9 with pre-diagnostic blood samples. One control was chosen at random for each lung cancer case from appropriate risk sets consisting of all cohort members alive and free of cancer (except non-melanoma skin cancer) at the time of diagnosis of the index case. Matching criteria included: date of birth (±1 year, relaxed up to ±5 years for cases without available controls), ethnicity, gender, date of blood collection (±1 month, relaxed up to±3 months, and further to ±6 months for cases without available controls) and detailed smoking status: never smokers, short-term former smokers (quitting smoking less than 10 years before blood draw), long-term former smokers (quitting smoking over 10 years before blood draw), current light smokers (<15 cigarettes/day at blood draw) and current heavy smokers (≥15 cigarettes per day at blood draw). After quality control, a total of 234 incident lung cancer cases (111 adenocarcinomas, 6 large cell cancers, 47 squamous cancers, and 29 small cell cancers) and 234 individually matched controls were available for this analysis. In the sample, the mean time from blood draw to diagnosis was 9.6 years (range: 1.1–17.5).

The EPIC is a large multicenter cohort study of diet and chronic diseases. The study rationale has been published previously^30^,^31^. In brief, in the EPIC Heidelberg cohort study (EPIC HD), 25,500 study participants from the general population were recruited from June 1994 to October 1998. Inhabitants of Heidelberg and of the surrounding region who met the age criteria of the EPIC study design (men: 40–64 years, women: 35–64 years) were randomly invited by mail to take part in the study. Study subjects were asked to complete questionnaires and were interviewed about their individual health, diet and lifestyle such as life history of tobacco smoking and alcohol intake. In addition, anthropometric measurements were taken and a blood sample of 30 ml was collected, which was fractionated and stored in aliquots in liquid nitrogen for future research. Up to six follow-up questionnaires were sent to the participants, at 2- to 3-year intervals, to ask about incident diseases and changes in lifestyle and diet. All self-reported incident cases of cancer were systematically verified against clinical and pathology records. The present study was based on 211 incident lung cancer cases identified by July 2015. Cases with <1 year from blood draw to diagnosis were excluded. Of the remaining cases those with the shortest follow-up times to diagnosis and who were either current or former smokers at the baseline recruitment were selected for this study (*n*=66). EPIC controls without any neoplastic disease were randomly matched to the lung cancer cases using an incidence density protocol. Matching was done on the basis of age at baseline (±5 years), gender, smoking status (current and former) and pack years (±1 PY). After initial quality control, 63 incident lung cancer cases (25 adenocarcinomas, 15 squamous cell carcinoma, 19 small-cell lung cancer and 4 uncharacterized lung cancers) with a mean interval between blood draw and diagnosis of 4.8 years (range: 1.1–8.6) and 63 individually matched controls remained for further analysis. This study was approved by the ethics committee of the Medical Faculty of the University of Heidelberg (S-627/2013).

### DNA methylation measurement and data pre-processing.

Genome-wide DNA methylation analyses were performed on pre-diagnostic blood samples using the Illumina Infinium HumanMethylation450 platform.

NOWAC laboratory procedures were carried out at the Human Genetics Foundation (Turin, Italy), using the Illumina Infinium HumanMethylation450 (HM450). Buffy coats stored in liquid nitrogen were thawed, and genomic DNA was extracted using the QIAGEN QIAsymphony DNA Midi Kit. 500 ng of DNA were bisulphite-converted using the Zymo Research EZ-96 DNA Methylation-Gold Kit, and hybridized to Illumina Infinium HumanMethylation450 BeadChips. These were subsequently scanned using the Illumina HiScanSQ system, and sample quality was assessed using control probes present on the micro-arrays. Finally, raw intensity data were exported from Illumina GenomeStudio (version 2011.1).

MCCS laboratory procedures were carried out at the Genetic Epidemiology Laboratory, the University of Melbourne according to manufacturers' protocols. DNA extraction from lymphocytes and buffy coats was performed using Qiagen mini spin columns, whereas dried blood spot DNA was extracted using a method developed in-house^32^ and the quality and quantity of DNA was assessed using the Quant-iT Picogreen dsDNA assay measured on the Qubit Fluorometer (Life Technologies). Samples were distributed into 96-well plates and processed in chips of 12 arrays (8 chips per plate) with case–control pairs arranged randomly on the same chip. All subsequent steps were performed as described above for NOWAC.

NSHDS laboratory procedures were carried out on two sites. DNA extraction from the buffy coat of EDTA-venous blood samples was conducted at the Umeå University, Sweden, using FlexiGene DNA Kit (QIAGEN GmbH). Illumina Infinium HumanMethylation450 BeadChip analysis was conducted at the ALSPAC/IEU Laboratory at the University of Bristol, according to the protocol described above for NOWAC.

EPIC HD laboratory procedures were carried out at the German Cancer Research Center (DKFZ) and at LGC Bioscience. Buffy coat DNA was isolated at LGC Bioscience by the company's standardized protocols and returned to DKFZ. DNA methylation profiling with the Illumina Infinium HumanMethylation450 BeadChip array was performed according to the manufacturer's instructions at the DKFZ Genomics and Proteomics Core Facility. Quality control of genomic DNA included three independent measurements with Quant-iT Picogreen dsDNA assay and all samples were tested on 1% agarose gels for DNA integrity. The Zymo Research EZ-96 DNA Methylation Kit was used for bisulfite conversion of DNA. All subsequent steps were performed as described for NOWAC.

NOWAC data pre-processing was carried out using in-house software written for the R statistical computing environment. For each sample and each probe, measurements were set to missing if obtained by averaging intensities over less than three beads, or if averaged intensities were below detection thresholds estimated from negative control probes. Background subtraction (to remove background noise) and dye bias correction (for probes using the Infinium II design) were also performed. The resulting subset of 473,929 probes targeting autosomal CpG loci was selected for further analyses, and among these, probes with missing values in more than 20% of the samples were excluded from the analyses, leaving 450,890 probes. Samples with more than 5% of non-detected probes were also excluded from the analysis (14 samples excluded).

For the MCCS, methylation data were normalized to the internal built-in controls as provided by the standard Illumina software and subset-quantile within array normalization for type I and II probe bias correction^33^. The 65 CpGs corresponding to single-nucleotide polymorphisms were excluded. Methylation measures were assigned as missing for CpG sites with a detection *P*-value higher than 0.01. No samples failed (a sample was considered as ‘failed' if more than 5% of the CpG measures were missing) and 182 (0.04%) CpG sites were excluded because values were missing for more than 20% of the samples, thus leaving 485,330 CpGs suitable for the analysis. Only the 458 male samples were considered when filtering probes in the Y chromosome.

In the NSHDS, methylation data were normalized using a functional normalization procedure that uses the built-in control probes to remove unwanted technical variation^34^. CpG sites that mapped to multiple genomic regions were excluded^35^. CpG sites with a detection *P*-value >0.01 were set to missing. CpG sites were excluded if they were missing in more than 20% of samples. Samples were excluded if more than 5% of their CpG sites were missing or if their average detection *P*-value was >0.01. Samples were also dropped if their case–control pair was missing. Of 490 samples initially available, 22 were excluded on the basis of the aforementioned procedures, leaving a total of 234 matched case–control pairs for analysis. Methylation levels at each locus were quantified using the beta-values^36^.

In EPIC HD, the quality control measures included removal of SNP-containing probes, removal of CpGs not analysed in all samples or those in non-CpG context, correction for batch effects and normalization with beta quantile dilation method: 63 sample pairs entered the final differential methylation analysis.

### Statistical analysis

*Association study*. In the NOWAC study, unconditional logistic regression models were used for all analyses, with DNA methylation levels included as an independent variable and standardized to 1 s.d. To account for residual technical confounding, all models were adjusted for micro-array and position of the sample on the micro-array. All analyses were additionally adjusted for blood cell composition differentials estimated using the algorithm developed by Houseman *et al.*^37^ by including in the model the percentage of each cell type. The Houseman prediction model was calibrated using DNA methylation profiles of purified human leukocytes from six healthy male blood donors, and predictions were obtained using the subset of 89,490 probes found to be differentially methylated across cell types at a stringent Bonferroni-corrected significance threshold ensuring a family-wise error rate below 0.01. Further adjustment included matching variables (year of birth, date of blood collection). Multiple testing was accounted for by using a stringent strategy: Bonferroni correction with control of the family-wise error rate below 0.05.

In NOWAC, we also built a predictive model based on smoking status and methylation of *AHRR* and *F2RL3,* and estimated the AUC with and without gene methylation. This was not feasible for the other cohorts because of matching by smoking.

In MCCS, conditional logistic regression was applied to estimate ORs of lung cancer. A stratified analysis by smoking status (never/former/current smokers) was also performed with further adjustment for number of cigarettes smoked (<15, 15–24, 25 or more per day), duration of smoking (<30 years, 30–39, 40 or more) and time since quitting (<5 years, 5–14, 15 or more). Associations between smoking and methylation levels were assessed by fitting linear-mixed effect models with random intercepts to the *M*-values of methylation (*M*=log2(beta/(1-beta)))^36^ with three levels of clustering due to matching sets being within batch and these within plate. The model was also controlled for the fixed effects of age at blood collection, gender and the smoking variables.

In the NSHDS, ORs for lung cancer were estimated by conditional logistic regression. Owing to the case–control matching, all models were adjusted for age, sex, smoking status (never/former/current smokers) and smoking quantity (1–14 versus >14 cigarettes per day) by design. To estimate the separate effects by smoking status, models were run separately for never, former and current smokers, with adjustment for time since quit smoking (in former smokers only) and smoking duration (in former and current smokers).

In EPIC HD, blood cell type composition of every sample was estimated^37^ using granulocytes, CD4^+^ and CD8^+^ T cells, natural killer cells and monocytes. A principal component analysis of the cell types was performed and the first two principal components were included in a linear regression model of methylation differences for every CpG. The risk of lung cancer was then modelled using conditional logistic regression on standardized residuals obtained from the cell type regression, adjusting for the average number of cigarettes smoked, duration of smoking and time since smoking cessation (for former smokers). Lung cancer risk was investigated using the overall study population as well as for subgroups determined by smoking status at baseline (current or former) and ORs and 95% CIs were computed.

*Mediation analysis*. We performed mediation analysis to assess whether methylation of cg05575921 (*AHRR*) and cg03636183 (*F2RL3*) probes mediated the effect of smoking (ever smoking versus never smoking) on lung cancer risk using parametric G-computation^38^ achieved by Monte Carlo simulations^39^ and adapted to deal with the case–control design following VanderWeele and Vamsteelandt^40^. This requires the specification of a model for the mediator and one for the outcome. Linear regression was used to model methylation levels as a function of smoking status, age and their interaction, and logistic regression to model lung cancer status as a function of age, smoking status, methylation and their interactions. The linear regressions for methylation were weighted to account for the study design; cases were weighted by the prevalence of lung cancer and controls were weighted by 1 minus the prevalence.

We quantified the amount by which either or both of the two methylation probes mediated the effect of smoking on lung cancer incidence by partitioning the TCE of smoking into a NIE and a NDE^41^,^42^. We expressed these quantities on the log OR scale because of the case–control design, although they can be interpreted as log rate ratios (because cases are incident lung cancers).

The NDE is the effect of smoking on lung cancer (on the log OR scale) when methylation takes the natural value it would have taken in the absence of smoking; whereas the NIE quantifies the change that would be found in log odds of lung cancer for smokers if we could change their methylation level to be that of never smokers. The TCE is the sum of these effects. The proportion of the total effect explained by the hypothesized mechanism (proportion mediated) is given by the ratio between NIE and TCE (on the log scale). Identification of the mediated proportion required structural and parametric assumptions, namely: no unmeasured exposure-mediator, mediator-outcome and exposure-outcome confounding; correct model specification for each of the outcome and the mediator(s)^41^,^42^.

In our analysis, it is possible that unmeasured confounders could lead to inaccurate estimates of the effects: in particular, regarding exposure-mediator confounders, information such as smoking intensity, duration of smoking and passive smoking would probably affect the final estimates. The ideal situation would be to create an exposure variable that summarizes all this information and to repeat mediation analysis using the new variable as the exposure variable. In our case, the presence of several missing values in NOWAC data prevented us from performing this type of analysis. Air pollution might be a confounder of the mediator-outcome relationship, but we assumed that it would be a negligible factor in Norway.

## Additional information

**How to cite this article:** Fasanelli, F. *et al.* Hypomethylation of smoking-related genes is associated with future lung cancer in four prospective cohorts. *Nat. Commun.* 6:10192 doi: 10.1038/ncomms10192 (2015).

## Supplementary Material

Supplementary Information Supplementary Tables 1-3

## Figures and Tables

**Table 1:** Top-ranked CpG sites for the locus-by-locus risk analysis in NOWAC data (discovery set): CpGs in the *AHRR* and *F2RL3* genes display the most significant inverse associations with risk (hypomethylation in cases).

**Probe name**	**Gene name**	**Chromosome**	**Position**	**Region**	**OR for 1 s.d.**	**95% CI**	***P*****-value**	***P*****-value Bonferroni**
cg05575921	*AHRR*	5	373378	N_Shore	0.37	0.31–0.54	3.33 × 10^−11^	1.36 × 10^−5^
cg03636183	*F2RL3*	19	17000585	N_Shore	0.40	0.31–0.56	3.86 × 10^−10^	1.58 × 10^−4^
cg21566642		2	233283329	Island	0.36	0.23–0.48	1.33 × 10^−9^	5.43 × 10^−4^
cg06126421		6	233284934		0.41	0.25–0.49	1.52 × 10^−9^	6.21 × 10^−4^
cg25305703	*CASC21*	8	233284402		0.45	0.35–0.60	3.28 × 10^−8^	1.34 × 10^−2^
cg21161138	*AHRR*	5	399360		0.46	0.36–0.62	5.01 × 10^−8^	2.04 × 10^−2^
cg01940273		2	26578098	Island	0.44	0.33–0.60	5.21 × 10^−8^	2.13 × 10^−2^
cg02451831	*KIAA0087*	7	30720080		0.43	0.29–0.57	6.55 × 10^−8^	2.67 × 10^−2^
cg05951221		2	233284661	Island	0.41	0.30–0.58	8.59 × 10^−8^	3.51 × 10^−2^
cg04884171	*BOLA2*	16	128378218	S_Shelf	0.33	0.15–0.41	1.18 × 10^−8^	4.82 × 10^−2^
cg03898802	*DOPEY2*	21	37617652	Island	0.37	0.29–0.57	1.20 × 10^−7^	4.90 × 10^−2^

CI, confidence interval; OR, odds ratio.

Unconditional logistic regression models were used with DNA methylation levels included as an independent variable and were adjusted for matching variables, micro-array, position of the sample on the micro-array and blood cell composition differentials.

**Table 2:** Results of the lung cancer risk analysis for the *AHRR* and *F2RL3* gene probes after strict adjustment for smoking in the discovery set and in the validation sets.

	**NOWAC**					**MCCS**				
	**ca**	**co**	**OR**	**95% CI**	***P*****-value**	**ca**	**co**	**OR**	**95% CI**	***P*****-value**
*AHRR cg05575921*										
Unadjusted	125	125	0.37	0.31–0.54	3.33 × 10^−11^					
Adjusted*	124	122	0.39	0.24–0.61	2.55 × 10^−05^	367	367	0.62	0.50–0.78	2.91 × 10^−5^
Never	11	54	0.90	0.26–3.10	8.70 × 10^−01^	43	43	0.63	0.24–1.64	3.47 × 10^−1^
Former†	41	33	0.23	0.10–0.56	1.00 × 10^−02^	153	153	0.48	0.31–0.75	1.45 × 10^−3^
Current‡	72	35	0.46	0.24–0.88	1.90 × 10^−02^	164	164	0.75	0.56–0.99	4.13 × 10^−2^
										
*F2RL3 cg03636183*										
Unadjusted	125	125	0.40	0.31–0.56	3.86 × 10^−10^					
Adjusted*	124	122	0.51	0.35–0.73	4.19 × 10^−04^	367	367	0.70	0.58–0.85	2.21 × 10^−4^
Never	11	54	1.07	0.29–4.00	9.20 × 10^−01^	43	43	0.78	0.44–1.36	3.73 × 10^−1^
Former†	41	33	0.25	0.35–0.55	1.00 × 10^−03^	153	153	0.70	0.50–0.98	3.81 × 10^−2^
Current‡	72	35	0.55	0.32–0.94	3.00 × 10^−02^	164	164	0.81	0.61–1.06	1.18 × 10^−1^

	**NSHDS**					**EPIC HEIDELBERG**				
	**ca**	**co**	**OR**	**95% CI**	***P*****-value**	**ca**	**co**	**OR**	**95% CI**	***P*****-value**
*AHRR cg05575921*										
Unadjusted										
Adjusted*	234	234	0.42	0.30–0.58	2.06 × 10^−7^	63	63	0.45	0.22–0.92	2.95 × 10^−02^
Never	26	26	1.96	0.40–9.68	1.10 × 10^−1^					
Former†	70	70	0.27	0.12–0.61	1.70 × 10^−3^	16	16	0.06	0.00–2.23	1.26 × 10^−01^
Current‡	120	120	0.47	0.31–0.72	5.40 × 10^−4^	47	47	0.56	0.27–1.16	1.16 × 10^−01^
										
*F2RL3 cg03636183*										
Unadjusted										
Adjusted*	234	234	0.61	0.47–0.79	1.56 × 10^−4^	63	63	0.62	0.38–1.04	7.02 × 10^−02^
Never	26	26	1.38	0.51–3.73	5.20 × 10^−1^					
Former†	70	70	0.45	0.26–0.80	6.60 × 10^−3^	16	16	0.29	0.03–3.24	3.17 × 10^−01^
Current‡	120	120	0.70	0.49–0.98	3.70 × 10^−2^	47	47	0.64	0.36–1.12	1.17 × 10^−01^

ca, case; CI, confidence interval; co, control; MCCS, Melbourne Collaborative Cohort Study; OR, odds ratio.

^*^In NOWAC, the estimates are from the unconditional logistic regression models adjusted for smoking status coded as never, former, current; in MCCS, the estimates are from the conditional logistic regression models where controls were matched on age, sex, date of blood collection, country of birth, type of biospecimen and smoking status as described in the text; in NSHDS, estimates are from conditional logistic regression models where cases and controls were matched on age, sex, smoking status and smoking quantity; in EPIC HD, the estimates are from conditional regression models where cases and controls were matched on smoking status and packyears of smoking.

^†^In MCCS and EPIC HD, the estimates are also adjusted for number of cigarettes smoked, duration of smoking and time since quitting smoking; in NSHDS, estimates are also adjusted for duration of smoking and time since quitting smoking.

^‡^In MCCS and EPIC HD, the estimates are also adjusted for number of cigarettes smoked and duration of smoking; in NSHDS, estimates are also adjusted for duration of smoking.

**Table 3:** Mediation analysis of the NOWAC cohort based on g-formula.

	**Log OR**	**s.e.**	***P-*****value**	**95% CI**
*AHRR-cg05575921*				
TCE	1.83	0.29	<0.001	(1.37–2.64)
NDE	1.26	0.31	<0.001	(0.75–2.08)
NIE	0.56	0.08	<0.001	(0.39–0.73)
Effect mediated	0.31	0.08	<0.001	(0.18–0.46)
				
*F2RL3-cg03636183*				
TCE	1.82	0.30	<0.001	(1.29–2.48)
NDE	1.23	0.33	<0.001	(0.63–1.93)
NIE	0.59	0.09	<0.001	(0.43–0.80)
Effect mediated	0.32	0.08	<0.001	(0.20–0.53)
				
*AHRR-cg05575921 and F2RL3-cg03636183*				
TCE	1.79	0.30	<0.001	(1.28–2.53)
NDE	1.13	0.34	0.001	(0.49–1.86)
NIE	0.66	0.15	<0.001	(0.42–1.09)
Effect mediated	0.37	0.11	0.001	(0.19–0.66)

CI, confidence interval; NDE, natural direct effect; NIE, natural indirect effect; OR, odds ratio; TCE, total causal effect.

TCE, NDE and NIE for the cg05575921 probe in *AHRR*, for the cg03636183 probe in *F2RL3* and for the two probes combined: 31% of the total effect of smoking on lung cancer risk is mediated by *AHRR* site-specific methylation, 32% of the total effect of smoking on lung cancer risk is mediated by *F2RL3* site-specific methylation and 37% of the total effect of smoking on lung cancer risk is mediated by the combined contribution of *AHRR* and *F2RL3* methylation (separate pathways for the two probes).
