# Supplementary material for: Hypomethylation of smoking-related genes is associated with future lung cancer in four prospective cohorts
Source: Nat Commun. 2015 Dec 15;6:10192. doi: 10.1038/ncomms10192 (PMC4682166; doi:10.1038/ncomms10192)
Supplement: Supplementary Information — Supplementary Tables 1-3 [file ncomms10192-s1.pdf]

| **Probe Name** | **Gene Name** | **Information** | **References** |
|---|---|---|---|
| cg05575921<br>cg21161138 | *AHRR* | *AHRR* mediates dioxin toxicity and is involved in regulation of cell growth and differentiation. | Zudaire E et al.<br>*The aryl hydrocarbon receptor repressor is a putative tumor suppressor gene in multiple human cancers.* |
| cg03636183 | *F2RL3* | *F2RL3* codes for the thrombin PAR-4<br>The function of PAR-4 is not fully clear yet but there is emerging evidence that it might be involved in the pathophysiology of several malignant tumors including lung cancer | Zhang Y et al.<br>*F2RL3 methylation, lung cancer incidence and mortality.* |
| cg21566642<br>cg01940273<br>cg05951221 | | the closest gene is *ALPPL2*<br>*ALPPL2* is a protein coding gene whose expression is strongly correlated with that of Heme Oxygenase-1 gene (it is expressed in many cancers and promotes growth and survival of neoplastic cells) | Tauber S et al.<br>*Transcriptome analysis of human cancer reveals a functional role of heme oxygenase-1 in tumor cell adhesion.* |
| cg06126421 | | the closest gene is *FLOT1*<br>*FLOT1* seems to have a role in non-small cell lung cancer tumorigenesis | Li H et al.<br>*Abnormal expression of FLOT1 correlates with tumor progression and poor survival in patients with non-small cell lung cancer.* |
| cg25305703 | *CASC21* | *CASC21* (cancer susceptibility candidate 21) has an oncogenic function | Kim T et al.<br>*Long-range interaction and correlation between MYC enhancer and oncogenic long noncoding RNA CARLo-5* |
| cg02451831 | *KIAA0087* | *KIAA0087* is a RNA Gene, and belongs to non coding RNA class<br>currently no evidence of involvement in cancer tumorigenesis | |
| cg04884171 | *BOLA2* | *BOLA2* encodes the BolA-like protein 2; this protein is conserved from prokaryotes to eukaryotes and seems to be involved in cell proliferation or cell-cycle regulation | Hunecke D et al.<br>*MYC-regulated genes involved in liver cell dysplasia identified in a transgenic model of liver cancer* |
| cg03898802 | *DOPEY2* | *DOPEY2* is a protein coding gene<br>currently no evidence of involvement in cancer tumorigenesis | |

**Supplementary Table 1 -** Main information about involvement in cancer pathways for the top-ranked CpGs found after the locus-by-locus risk analysis in NOWAC data (discovery set).

| | | ca | co | OR for 1 SD |
|---|---|---|---|---|
| ***cg05575921-AHRR*** | all | 125 | 125 | 0.37(0.31-0.54) |
| | time to diagnosis <5 years | 84 | 125 | 0.20(0.10-0.37) |
| | time to diagnosis >=5 years | 41 | 125 | 0.42(0.30-0.56) |
| | | **heterogeneity** | | p=0.021 |
| ***cg03636183-F2RL3*** | all | 125 | 125 | 0.40(0.31-0.56) |
| | time to diagnosis <5 years | 84 | 125 | 0.32(0.19-0.54) |
| | time to diagnosis >=5 years | 41 | 125 | 0.42(0.30-0.57) |
| | | **heterogeneity** | | p=0.375 |

**Supplementary Table 2 -** Analysis stratified by time to diagnosis.

| | cohorts | | | |
|---|---|---|---|---|
| | NOWAC | MCCS | NSHDS | EPIC-HEIDELBERG |
| **number of eligible cases** | 132 | 367 | 245 | 66 |
| **number of cases considered in the analysis*** | 125* | 367* | 234* | 63 |
| | **nested case-control studies** | | | |
| **age at baseline (years)** | 47 (range: 34 -61) | 59 (range: 39 - 70) | 55 (range: 29-64) | 56 (range: 39-65) |
| **age at diagnosis (years)** | 56 (range: 47 - 64) | 69 (range: 48 - 80) | 64 (range: 42-81) | 61 (range: 45-70) |
| **time from blood draw to diagnosis (years)** | 3.88 (range: 0.29 - 7.92) | 9.38 (range: 0.01 - 18.67) | 9.6 (range: 1.1-17.5) | 4.8 (range: 1.1-8.6) |
| **women (N)** | 250 | 276 | 230 | 22 |
| **men (N)** | 0 | 458 | 238 | 104 |

**Supplementary Table 3 -** Summary of the key characteristics of the study groups.
